# Clinical features and genetic analysis of 5 cases of infantile-type glycogen storage disease type II: Case reports

**DOI:** 10.1097/MD.0000000000039534

**Published:** 2024-08-30

**Authors:** Qi Feng, Meng Qiao Zhang, Chun Xiao Ba, Ying Qian Zhang

**Affiliations:** a Hebei North University, Zhangjiakou, Hebei, China; b Three Departments of respiration, Hebei Children’s Hospital, Shijiazhuang, Hebei, Chinac Hebei Medical University, Shijiazhuang, Hebei, China; c Hebei Medical University, Shijiazhuang, Hebei, China.

**Keywords:** glycogen storage disease type II, hypertrophic cardiomyopathy, infant

## Abstract

**Objective:**

Clinical and genetic mutation analysis was performed on 5 infantile glycogen storage disease type II children in Chinese mainland.

**Methods:**

Clinical data of 5 children with infantile-type glycogen storage disease type II due to the acidic α-glucosidase (*GAA*) gene variants diagnosed and treated at Hebei Provincial Children’s Hospital from January 2018 to April 2020 were retrospectively analyzed.

**Results:**

Among the 5 cases, 1 was female and 4 were male, and the age at first diagnosis was from 2 months to 7 months. The first symptoms of all 5 cases showed progressive muscle weakness, hypotonia, and motor developmental backwardness, and all of them had abnormally elevated creatine kinase, and the echocardiograms suggested different degrees of myocardial hypertrophy, with ejection fractions ranging from 44% to 67%. Analysis of *GAA* gene variations: all 5 cases were compound heterozygous, and a total of 12 variant loci were detected, of which c.2024_2026delACA, c.2853G > A, c.1124G > T, c.574G > A, c.2509C > T, and c.2013G > A were new mutations that had not been reported.

**Follow-up:**

All 5 children died before 1 year of age, and the age of death ranged from 7 months to 11.5 months, with a mean survival time of 9.8 months.

**Conclusion:**

Peripheral blood *GAA* gene testing and alpha-glucosidase enzyme activity testing is an effective method for diagnosing this disease.

## 1. Introduction

Glycogen storage disease type II (and it is abbreviated as GSD II) is a rare autosomal recessive disorder caused by mutations in the lysosomal endo-acidic alpha-glucosidase (*GAA*) gene. Neuromuscular diseases are characterized by progressive muscle weakness and skeletal muscle atrophy. Based on the age of onset, disease progression, and degree of organ involvement, GSD II can be divided into infantile and late forms. The infantile form has an early onset, usually starting at 2 to 3 months of age, with clinical symptoms such as unexplained elevation of serum creatine kinase, difficulty in feeding, a large tongue, muscular weakness, developmental delays, cardiac enlargement, left ventricular hypertrophy, and a poor prognosis, with most deaths occurring before the age of 1 year due to cardiorespiratory insufficiency. Late-onset forms have a late onset of the disease, most often after the age of 1 year.^[1]^ It is diagnosed at any age, with a slow rate of progression and less obvious clinical symptoms, making it more difficult to confirm the diagnosis. Currently, there are few reports of genetic studies on GSD II in children in China. Liu et al^[2]^ analyzed the cases in China and found that the diagnosis of GSD II was mainly based on *GAA* gene testing (69.7%), with about 33% of the cases undergoing myocardial biopsy, 16.2% of the cases undergoing *GAA* activity testing of skin fibroblasts, and 48.5% of the cases undergoing *GAA* activity testing of dried blood filtration paper slices and peripheral blood leukocytes; in this paper, we report 5 cases of GSD II diagnosed with *GAA* gene analysis and confirmed *GAA* gene testing. Clinical and genetic features of children with GSD II were confirmed by *GAA* gene analysis, and 5 new mutations were identified.

## 2. Information and methods

### 2.1. Subjects

Case 1: A 7-month-old male child was admitted to the hospital with a 3-month history of weight loss, fever, and dyspnea for 1 day. The child was born in the first trimester with a birth weight of 3.0 kg. He was born prematurely, with developmental delay, to a mother with gestational diabetes mellitus and hypertension. The father was physically fit, non-consanguineous, and denied any family history of hereditary diseases. The child was diagnosed with hypertrophic cardiomyopathy at the age of 6 months in our hospital and was given symptomatic supportive treatments such as myocardial nourishment and improvement of cardiac function, but the effect was unsatisfactory. One day prior, he developed fever and dyspnea, and was readmitted to the hospital again. Physical examination revealed a clear, poor spirit, no spontaneous respiration, dull heart sounds, liver 3 cm below the ribs, limb muscle strength grade II, and no deformity of the spine and limbs. The laboratory test results were as follows: ALT, 179 U/L (5–40 U/L); AST, 390 U/L (5–40 U/L); CK, 1301 U/L (24–194 U/L); CK-MB, 64 IU/L (0–25 IU/L); and N-terminal brain natriuretic peptide, 300 pg/mL (<200 pg/mL). Laryngoscopy, left vocal cord paralysis; chest X-ray, large heart shape; echocardiography revealed ejection fraction (EF) of 67%, ventricular septum and left ventricular posterior wall thickening; endocardial echogenicity enhancement; ventricular septum end-diastolic thickness of 11 mm; and left ventricular posterior wall end-diastolic thickness of 13 mm. After admission, the child was actively engaged in cardiopulmonary resuscitation, mechanical ventilation, rehydration, acid correction, glucose lowering, cardioplegic, and anti-infective measures. However, the child’s circulatory failure gradually worsened, and the blood pressure could not be maintained, which was not significantly improved by combined dopamine, milrinone, and epinephrine hydrochloride treatments. Malignant cardiac arrhythmia occurred after 2 days of bi-level positive airway pressure (BiPAP) treatment. Glycogen storage disease (GSD) type II infantile type may present with respiratory insufficiency in order to clarify the diagnosis of the proposed *GAA* activity test or *GAA* gene analysis.

Case 2: A 3-month-old male child was admitted to the hospital with liver function abnormalities for 2 months and cough for 10 days. The child was born at full term in the second trimester of the third pregnancy, with a birth weight of 3.9 kg. The parents were healthy and not consanguineous, and denied any family history of genetic predisposition. At present, the child’s head is unstable, cannot turn over, and their growth and development lags behind that of normal children of the same age. Physical examination revealed a malnourished appearance, chronic disease appearance, and clear consciousness. No yellowing of the skin or sclera was observed. There was no cranial deformity, nasal agitation, slight cyanosis of the lips and mouth, or a weakly positive triple concave sign. Breath sounds were coarse in both lungs and fine wet rhonchi could be heard. There was no elevation in the precordial region, heart rate was 150 beats per minute, rhythmic, heart sounds were low and dull, and there was no murmur. The abdomen was soft, the liver was 3 cm below the right ribs with a medium texture, and the spleen was not palpable below the left ribs. The muscle strength of both lower limbs was grades 3 to 4, with hypotonia and no deformities of the spine and limbs. Laboratory tests were as follows: ALT, 203 U/L (5–40 U/L); AST, 349 U/L (5–40 U/L); CK, 823 U/L (24–194 U/L); CK-MB, 68 IU/L (0–25 IU/L); N-terminal brain natriuretic peptide, 382 pg/mL (<200 pg/mL); EBV (+), *Chlamydia pneumoniae* IgM antibody (+), and respiratory syncytial virus IgM antibody (+). Cranial MRI: bilateral frontotemporal extracerebral space widening. Laryngoscopy: left vocal cord paralysis. Chest CT revealed inflammation of both lungs, hypodensity of cardiac chambers, and thickening of the myocardium. ECG: ST-T segment changes. Echocardiography revealed EF64% and myocardial thickening of the right and left ventricles with enhanced echogenicity. The left ventricular end-diastolic diameter was 22 mm, septal end-diastolic thickness was 10 mm, left ventricular posterior wall end-diastolic thickness was 9 mm, and right ventricular free wall myocardium was approximately 6.7 mm thick. Abdominal ultrasonography revealed that the echogenicity of the liver parenchyma was slightly thickened and inhomogeneous, and the gallbladder wall was rough and slightly thick. After admission, cefepime, azithromycin, ribavirin were given to fight infection, propranolol hydrochloride to reduce myocardial oxygen consumption, but the effect was not good, the diagnosis was considered glycogen storage disease, and glycogen storage disease subtypes in the GSD II infantile type can be seen in recurrent respiratory tract infections, so in order to further clarify the diagnosis and type of *GAA* activity test or *GAA* gene test is proposed to be carried out.

Case 3: A 6-month-old male child was admitted to the hospital with “growth retardation and elevated liver enzymes for 3 days.” The child was born at 41 weeks of gestation in the second trimester with a birth weight of 3.8 kg. The mother’s blood pressure and glucose levels were low during pregnancy, and the child had poor milk intake after birth. Physical examination: the child was lagging behind his peers in growth, poorly nourished, had a normal face, and was in a clear state of mind. There was no yellowing of the skin or sclera and the superficial lymph nodes were not palpable. Respiratory sounds were coarse in both lungs and no dry or wet rhonchi was detected. The heart rate was 140 beats/min, with synchronized heart rate, strong heart sounds, and no murmurs. The abdomen was flat and soft, the liver was soft and 1 cm below the right rib, and the spleen was not palpable below the left rib. There was no deformity of the spine and limbs, no hypertrophy of the gastrocnemius muscle, muscle strength of both lower limbs in grades 3 to 4, hypotonia, negative Gower sign, tendon reflexes were present, and pathological signs were all negative. Laboratory tests were as follows: ALT, 122 U/L (5–40 U/L); AST, 206 U/L (5–40 U/L); CK, 605 U/L (24–194 U/L); CK-MB, 70 IU/L (0–25 IU/L); and N-terminal brain natriuretic peptide level, 345 pg/mL (<200 pg/mL). Cranial MRI: bilateral frontotemporal extracerebral space widening. Chest radiograph: the heart shadow is slightly larger. Electrocardiography revealed sinus tachycardia, incomplete right bundle branch block, Q waves in some leads, and left ventricular hypervoltage. Echocardiography revealed EF61%, septal and left ventricular wall thickness, left ventricular endocardial thickening, echo enhancement, lax and thickened myocardial tissue structure of the apical part of the left ventricle, a small amount of bicuspid and tricuspid regurgitation, and reduced contractile function of the left heart. End-diastolic thickness of the interventricular septum was 10 mm, and end-diastolic thickness of the posterior wall of the left ventricle was 8 mm. Neurologic EMG: both eyes were stimulated separately, waveform differentiation was poor, and latency was approximately normal. After admission, he was administered ceftriaxone tazobactam sodium and amoxicillin to fight the infection, captopril, and propranolol to reduce myocardial oxygen consumption, with unsatisfactory effects, and the diagnosis was considered a genetic metabolic disease. One of the GSD II infantile types mainly showed progressive muscle weakness, hypotonia, and backward motor development, and laboratory tests revealed that myosin and liver enzymes were increased. Therefore, to further define the diagnosis and type of *GAA* activity, a *GAA* gene test was proposed.

Case 4: A 2-month-old male was admitted to the hospital with “intermittent phlegm in the throat for more than 20 days, aggravated for 9 days.” The child was born in the first trimester, with a birth weight of 3.0 kg, premature, and developmentally delayed. Parents are healthy, non-consanguineous marriages, and deny family genetic history. Physical examination: development is behind the same age, poor nutrition, normal face, and a clear mind. There was no yellow staining of the skin and mucous membranes of the entire body, and the superficial lymph nodes were not palpable. Breath sounds were coarse in both lungs and no dry or wet rhonchi was detected. Heart rate was 150 beats per minute, unison, strong heart sound, and no murmur. The abdomen was flat and soft, the liver was soft and 1 cm below the right ribs, and the spleen was not palpable below the left ribs. The right side of the tongue was hypertrophied, with an open mouth and extended tongue, 6-finger deformity of the left hand, palm of the right hand through the palm, and symmetrical dermatoglyphics of both lower limbs. There was no spinal deformity, no hypertrophy of the gastrocnemius muscle, muscle strength of both lower limbs in grades 3 to 4, hypotonia, Gower sign was negative, tendon reflexes were present, and pathological signs were all negative, calf circumference of the left side was 20 cm, and calf circumference of the right side was 18 cm. Laboratory tests: ALT 120 U/L (5–40 U/L), AST 150 U/L (5–40 U/L), CK 300 U/L (24–194 U/L), CK-USD 300 U/L (24–44 U/L), and AST 150 U/L (5–40 U/L), (U/L) CK-MB, 63 IU/L (0–25 IU/L); and N-terminal brain natriuretic peptide, 365 pg/mL (<200 pg/mL). Cranial MR: bilateral frontotemporal brain widening of the external interstitial glands and bilateral ventricular widening of the temporal and anterior horns. Laryngoscopy revealed congenital laryngeal cartilage softening and cyst of the tongue root. Neck CT: relative hypodensity of the right lobe of the thyroid gland and fullness of the thymus. Chest CT: uneven translucency in both lungs, multiple patchy shadows in both lungs, inflammation, narrowing of the thoracic trachea, and large thymus. Chest enhanced CT: bilateral congenital pulmonary airway malformations, right lung developmental anomalies, mid-main airway stenosis. Echocardiography revealed EF61%, septal end-diastolic thickness 5 mm, and left ventricular posterior wall end-diastolic thickness 4 mm. After admission to the hospital to reduce myocardial oxygen consumption by anti-infection, propranolol, the effect was unsatisfactory, and genetic metabolic disease was diagnosed. Physical examination of the child suggested glycogen storage disease, so to further clarify the diagnosis and type of *GAA* activity test or *GAA* gene test was proposed.

Case 5: Female, 7 months was admitted to the hospital with “poor mental health, refusing breastfeeding for 3 days, and fever for 1 day, and was born in the first month of her first pregnancy, delivered by cesarean section at full term, with a birth weight of 3.7 kg. The patient’s growth and development were uneventful. His parents were healthy, not consanguineous, and denied any family history of hereditary disease. Physical examination revealed a poor spirit, no spontaneous respiration, signs of malnutrition, and thin subcutaneous fat. Heart sounds were low and dull, the liver was 3 cm below the ribs, muscle strength of the limbs was reduced, the extremities were cold and wet, and the feet were mildly edematous. Laboratory tests were as follows: ALT, 99 U/L (5–40 U/L); AST, 260 U/L (5–40 U/L); CK, 1120 U/L (24–194 U/L); CK-MB, 100 IU/L (0–25 IU/L); and N-terminal brain natriuretic peptide, 980 pg/mL (<200 pg/mL). Chest radiograph: exudative shadow in the left lung and cardiomegaly. Electrocardiography revealed tachycardia. Echocardiography revealed EF44%, septal end-diastolic thickness 9 mm, left ventricular posterior wall end-diastolic thickness 10 mm, left ventricular enlargement, septal and left ventricular wall myocardial thickening, echo enhancement, and left ventricular systolic function were reduced. Abdominal ultrasound: hepatic size, 3 cm below the ribs. Parenchymal echogenicity was slightly thickened and less homogeneous. After admission, the child was actively undergoing cardiopulmonary resuscitation, mechanical ventilation, rehydration, acid correction, hypoglycemia, cardioplegia, and anti-infection therapy. The child’s circulatory failure progressively worsened, blood pressure could not be maintained, and was treated with combined dopamine, milrinone, and epinephrine hydrochloride without significant improvement. The diagnosis was a genetic metabolic disease. The child’s indicators suggested glycogen storage disease, so to further define the diagnosis and type of *GAA* activity test or *GAA* gene test was proposed.

### 2.2. Further examination

The study was reviewed and approved by the hospital’s Ethics Committee and written informed consent was secured from the legal guardian. Peripheral blood (5 mL) and their parents were extracted, and 20 exons of the *GAA* gene (NM_000152.4) and their neighboring introns were sequenced and analyzed using whole-genome sequencing, which was performed in accordance with the American College of Medical Genetics and Genomics (American College of Medical Genetics and Genomics). The Medical Genetics and Genomics (ACMG) guidelines were also evaluated. HGVS nomenclature was used. Genetic variants that were clearly or potentially associated with the clinical phenotype of the subjects were verified using Sanger sequencing.

## 3. Results

### 3.1. Genetic test results

Sequencing results revealed that all 5 children were heterozygous for *GAA* gene variants, and 12 different variant loci were detected (Table 1), including c.2024_2026delACA (p.N675del) minor deletion mutation, c.118C > T (p.R40X), c.2853G > A (p.W951X), and c.2237G > A (p. W746X) 3 nonsense mutations, of which c.2853G > A (p.W951X) was not reported in the literature, c.875A > G (p.Y292C), c.1669A > T (p.I557F), c.2132C > G (p.T711R), c.1124G > T (p.R375L), c.574G > A (p. E192K), c.2509C > T (p.R837C), c.1634C > T (p.P545L), and c.2013G > A (p.M671I) 8 missense mutations, including c.2132C > G (p.T711R), c.1124G > T (p.R375L), c.574G > A (p.E192K), c.2509C > T (p.R837C), c.2013G > A (p.M671I) were not reported in the literature.

**Table 1:** Genetic test results of 5 children.

Cases	Gender	Nucleotides – amino acids	Source of variation	Variation type	Pathogenicity analysis	Whether this variant is included in the gene bank
1	Male	c.875A > G(p.Y292C) c.2024_2026delACA(p.N675del)	Paternal source Mother source	Missense –	LP VUS	Yes No
2	Male	c.118C > T (p.R40X) c.1669A > T (p.I557F) c.2132C > G (p.T711R)	Paternal source Mother source Mother source	Nonsense Missense Missense	P VUS VUS	Yes Yes No
3	Male	c.2853G > A (p.W951X) c.1124G > T (p.R375L)	Paternal source Mother source	Nonsense Missense	LP LP	No No
4	Male	c.574G > A (p.E192K) c.2509C > T (p.R837C)	Paternal source Mother source	Missense Missense	VUS VUS	No No
5	Female	c.1634C > T (p.P545L) c.2013G > A (p.M671I) c.2237G > A (p.W746X)	Mother source Mother source Paternal source	Missense Missense Nonsense	P VUS P	Yes No Yes

VUS = variant of uncertain significance; LP: likely pathogenic; P: pathogenic.

### 3.2. Treatment and prognosis

Five children were not treated with enzyme replacement therapy because of economic reasons. Five children were hospitalized during the period of Cases 1 to 4 were admitted to the hospital after active anti-infective, liver protection, myocardial nutrition, and other symptomatic treatment; the treatment effect was poor; the parents gave up the treatment of the hospital, and after discharge from the hospital, Telephone follow-up after discharge, Cases 1 to 4 of the children were found to alpha-glucosidase enzyme activity in other hospitals was lower than the normal value. All died before the age of 1 year, the age of death was 7 months to 11.5 months, with an average survival time of 9.8 months. Case 5 was admitted to the hospital and discharged because of heart failure and failure to rescue.

## 4. Discussion

GSD II is caused by a lack of *GAA* activity due to mutations in the *GAA* gene, which leads to the inability to decompose glycogen, resulting in the manifestation of lysosomal degradation disorders of glycogen deposition in cardiac muscle, skeletal muscle, smooth muscle, and other systemic organs. According to survey statistics, the incidence of this disease is 1/100,000 to 1/14,000 in foreign countries and 1/17,000 in Taiwan and China.^[3]^ In China, the disease is often underdiagnosed and misdiagnosed owing to incomplete epidemiologic data, clinical rarity, and less awareness of the disease among clinicians. Type II glycogen accumulation disorder can be categorized into infantile and advanced forms. The infantile form of the disease causes multisystemic involvement and functional impairment of organs including the heart and respiratory muscles. Infants develop elevated creatinine kinase levels, hypertrophic cardiomyopathy, growth disorders, hypotonia, and generalized muscle weakness in the first 6 months of life, and the disease progresses rapidly, with most untreated children dying of respiratory and cardiac muscle involvement by the age of 1 year. In late-onset cases, the disease progresses at a slower rate with simple limb weakness.^[4]^ In this study, 5 children were not treated with enzyme replacement-specific therapy and were only treated with conventional symptomatic therapy, which was ineffective, and all of them died before the age of 1 year. Five children were diagnosed with infantile GSD II using a combination of genetic testing and clinical symptoms.

Glycogen storage can cause myocardial damage and hypertrophy, and studies have found that the most prominent clinical manifestations in children with infantile-type GSD II are cardiac insufficiency and progressive myocardial hypertrophy.^[5,6]^ In this study, chest radiographs and echocardiograms of 5 children with *GAA* suggested that the myocardium showed thickening of varying degrees, and the left ventricular ejection fraction ranged from 44% to 67%. Lukacs et al found that creatine kinase has a high sensitivity and is often elevated by a factor of 4 to 10, and suggested that for nonspecific persistent and unexplained elevation of CK, an early genetic test for *GAA* is more meaningful than muscle biopsy.^[7,8]^ The diagnosis of II relies on enzyme activity assays, muscle biopsies, and genetic testing.^[9]^ Most patients with *GAA* have ECG and echocardiographic abnormalities, and 80% of children with *GAA* may have ventricular hypertrophy with wall infiltration, shortened PR intervals, and QRS waves, which may be associated with abnormalities of the conduction system with enlargement of specialized conduction tissue cells with glycogen and deformation of the apical septal hypertrophy. These conduction abnormalities, combined with the hypertrophic cardiomyopathy characteristic of Pompe disease, put these patients at a high risk for tachyarrhythmias and sudden death.^[10,11]^ In this study, the ECGs of 3 children with *GAA* were dominated by shortened PR intervals and wide aberrant QRS waves as well as ST-T segment changes and abnormal sinus tachycardia.

GSD II is an autosomal recessive metabolic muscle disease caused by a double allele mutation in the *GAA* gene on chromosome 17q25.^[12]^ According to the *GAA* variant database (http://www.pompevariantdatabase.nl/) (data updated to October 2023), 910 variant loci of the *GAA* gene have been identified, the most common being mutations at the c.-32-13T > G locus. Of these, 620 (68.1%) were pathogenic variants of varying severity, including severely pathogenic, probably not severely pathogenic, not severely pathogenic, and probably mildly pathogenic; whereas a total of 290 (31.9%) were probably not pathogenic, not pathogenic, and pathogenicity unspecified variants. As the *GAA* gene has become increasingly recognized, genetic diagnostic methods have become crucial for the early diagnosis of *GAA*. There is a clear ethnic specificity of the *GAA* gene mutation. The c.118C > T mutation in the child in case 2 was found to be severely pathogenic in a Japanese study by Fukuhara^[13]^ as a common infantile-type GSD II genotype in the Japanese region. The c.1634C > T and c.2013G > A compound heterozygous mutations in the child in Example 5 have been shown to be pathogenic and are common infantile GSD II genotypes in the Netherlands. The c.2024_2026delACA microdeletion mutation and the c.875A > G missense mutation in the child of Example 1 have been reported in the literature^[14,15]^ to be possible non-severe disease-causing mutations, which occur in infantile GSD II. The child in Example 1 had a missense mutation at this locus and may also interact with a compound heterozygous mutation with the c.2024_2026delACA mutation to cause the clinical phenotype. The c.2853G > A and c.1124G > T mutations in the child in case 3 were the result of the combined effect of a heterozygous mutation from nucleotide 2853, guanine G, to adenine A, which resulted in an amino acid nonsense mutation, and a missense mutation due to the change of amino acid 375 from arginine to leucine, respectively, leading to the appearance of the clinical phenotype; however, the pathogenicity of this mutation has not been reported in the literature, and it is likely to be due to the transformation of the basic-carrying. The conversion of arginine to the neutral amino acid leucine, which affects the synthesis of *GAA*, is not a polymorphic change and occurs very infrequently in the population; thus, the c.2853G > A nonsense variant and the c.1124G > T missense mutation may be a new pathogenic mutation. In case 4, c.574G > A and c.2509C > T compound heterozygous mutations resulted in a change in amino acid 192 from glutamic acid to lysine and amino acid 837 from arginine to cysteine, respectively. The pathogenicity of this mutation has not been reported in the literature and may be due to a decrease in *GAA* enzyme activity due to the change from acidic glutamate to basic lysine and basic arginine to the neutral amino acid cysteine. Five children with *GAA* gene pathogenicity, classified as variant of uncertain significance, died within the first year of follow-up. We suspect that this mutation may be a pathogenic variant, and its pathogenicity classification may change in the future as the pathogenicity classification of this mutation has been studied.

GSD II is a disease with systemic multisystem involvement that requires multidisciplinary and individualized care plans for intervention.^[16]^ With further developments in medical technology, this disease has become a treatable rare disease. Early diagnosis and treatment are key to improving prognosis. Current GSD II treatments include nonspecific and specific treatments.^[17]^ Nonspecific treatment modalities include artificial ventilation, physiotherapy, and nutritional support. The most classical of the specific treatments is enzyme replacement therapy, KISHNANI P S et al^[18,19]^ found that early and standardized use of enzyme replacement therapy resulted in almost 100% survival of the child at 1 year of age, which could be maintained at approximately 89% at 2 years of age and 39% at 3 years of age. In contrast, the 18-month survival rate of children who were not administered enzyme replacement therapy is only 12.3%. The above data suggest that the use of enzyme replacement therapy may lead to an increase in the survival rate of children; the earlier it is used, the higher the survival rate. However, the basic conditions of the 5 children in this report were poor, and the enzyme replacement therapy treatment required millions of dollars per year and was not a health insurance reimbursement disease; therefore, the families of the children gave up the specific enzyme replacement therapy for comprehensive consideration. The 5 children in this study were not treated with enzyme replacement therapy, and the mortality rate reached 100% before 1 year of age, which is consistent with a previous report. Therefore, it is recommended that the relevant governmental departments should strengthen early screening and diagnosis, promote the improvement of the national rare disease diagnosis and treatment capacity, facilitate research, and increase the supply of generic drugs with higher quality and efficacy by pharmaceutical companies, so as to reduce medical costs and better meet public demand. Pharmaceutical companies are encouraged to produce generic drugs that are essential for clinical treatment and in short supply, especially for the treatment of major infectious diseases, rare diseases, pediatric diseases, and public healthcare crises. The state should implement a value-added tax policy for certain rare disease drugs to reduce the financial burden on the patients and their families.

However, some studies suggest that with the single use of enzyme replacement therapy, the therapeutic effect is poor, and there are more side effects.^[20]^ Recently published studies^[21,22]^ have also identified therapies that can be used both as a means to improve the effectiveness and tolerability of enzyme replacement and as stand-alone and revolutionary emerging treatments for several genetic diseases such as: chaperone therapy, gene therapy, autologous hematopoietic cell transplantation in combination with lentiviral therapy, oligonucleotide therapy, and substrate reduction, which have all Promising results were obtained, but these treatments have not been validated in humans.^[23]^ In summary, we suggest that early improvement of genetic testing for children with symptoms such as growth retardation, hepatomegaly, and tongue hypertrophy will help to clarify the diagnosis and provide a scientific basis for genetic counseling for children with GSD. However, it is crucial to acknowledge the limitations of this study. This suggests different mutational origins in the Chinese population as compared to geographically proximate countries, as well as genetic heterogeneity, although more patients need to be studied.

## Author contributions

**Conceptualization:** Qi Feng.

**Data curation:** Qi Feng.

**Formal analysis:** Qi Feng.

**Investigation:** Meng Qiao Zhang, Chun Xiao Ba.

**Methodology:** Meng Qiao Zhang, Chun Xiao Ba.

**Resources:** Meng Qiao Zhang, Chun Xiao Ba.

**Software:** Ying Qian Zhang, Meng Qiao Zhang.

**Supervision:** Chun Xiao Ba.

**Writing – original draft:** Qi Feng.

**Writing – review & editing:** Qi Feng, Ying Qian Zhang.
